# Factors associated with mortality among patients with culture-positive pulmonary tuberculosis in the urban poor population of Osaka City, Japan

**DOI:** 10.5365/wpsar.2021.12.3.836

**Published:** 2021-09-16

**Authors:** Akira Shimouchi, Yuko Tsuda, Jun Komukai, Kenji Matsumoto, Hideki Yoshida, Akihiro Ohkado

**Affiliations:** a Nishinari District Public Health Office, Osaka City, Japan.; b The Research Institute of Tuberculosis, Japan Anti-Tuberculosis Association, Japan.; c Osaka City Public Health Office, Japan.; d Faculty of Health and Nutrition, Otemae University, Japan.

## Abstract

**Objective:**

To determine the characteristics associated with mortality in patients with culture-positive pulmonary tuberculosis (PTB) in Airin, Osaka City, Japan.

**Methods:**

The characteristics of patients with culture-positive PTB registered between 2015 and 2018 in Airin, Osaka City, Japan, were compared between those who died of all causes before or during treatment and those who completed treatment.

**Results:**

Of the 241 culture-positive PTB patients eligible for this study, 170 completed treatment, with negative sputum culture tests, and 62 died. The all-cause case fatality rate was 26.7% (62/232). Multivariate analysis showed that mortality was associated with age ^3^70 years, having a positive sputum smear, a body mass index of < 18.5 and serious comorbidities such as cancer and heart and renal disease. Detection of tuberculosis (TB) by screening or in an outpatient department (OPD) for other diseases was inversely associated with mortality.

**Discussion:**

Detection of PTB by chest X-ray screening and during regular visits to OPDs for other diseases was associated with non-fatal TB and might contribute to early case finding. Therefore, current active TB case finding and health education on regular visits to physicians for other diseases should be strengthened further for the urban poor population of Osaka City, Japan.

A key milestone of the World Health Organization’s End TB Strategy is a 75% reduction in deaths from tuberculosis (TB) between 2015 and 2025 worldwide. (*1*) To achieve this, the annual decrease in the estimated global incidence rate of TB (110 per 100 000 population in 2015) should be accelerated from 2%/year in 2015 to 10%/year in 2025, and the case fatality rate among TB patients should be reduced from 15% in 2015 to 6.5% in 2025. (*1*)

In Japan in 2018, the TB incidence rate was 12.3 per 100 000, corresponding to an annual reduction of 6% from 2013. The TB case fatality rate in 2017 was 22.5% in all age groups but was higher for patients aged (*2*)70 years (34.6% vs 4.9% for patients aged 0–69 years). (*2*) Neither indicator meets the global targets.

In 2018, Osaka City had the highest TB notification rate of all cities in Japan, at 29.3 per 100 000 population, and the highest rate (298 per 100 000) was in the small, densely populated area of Airin (21 500/0.62 km^2^). (*3*) The annual reduction in the TB incidence rate in Airin during 2013–2018 was 5%, (*3*) similar to the national average. The TB case fatality rate in Airin during 2015–2018 was 25.9% for all age groups, 15.5% for patients aged 0–69 years and 41.7% for patients aged (*2*)70 years. (*3*)

Airin was a residential area during the country’s period of economic growth between the 1950s and the 1980s, accommodating Japan’s largest population of day labourers, including factory, dock and construction workers. Its peak population was in 1960, when there were 30 306 residents. (*4*) During the economic recession in 1993, however, many lost their jobs, some became homeless and the influx of workers into Airin ceased. The number of day labourers covered by insurance fell by 91.7%, from 24 458 in 1986 to 2025 in 2009. (*4*) As a result, the population has aged, the percentage of the population aged (*2*)65 years increasing from 8 to 10% in 1975–1990 to 20% in 2000, 30% in 2005, 40% in 2010 (*4*) and 45% in 2015. (*3*) Airin has the highest proportion of population aged (*2*)65 years in Osaka City, where the city average was 25% in 2015. (*5*)

In 2015, the population of Airin was 21 500; 81% were male, 52% were pensioners or day labourers, 43% were in a public assistance programme (PAP) that provides free medical services and 5% were homeless. In comparison, 6% of the population of Osaka City are in a PAP. More than half of all homeless people in Osaka City live in Airin. (*3*, *5*) Consequently, the population of Airin has become the oldest and the poorest in Osaka City.

To reduce the high TB notification rate in Airin, the community TB control programme has strengthened active case finding through chest X-ray screening and directly observed treatment (DOT) since 2013. (*3*) Active case finding comprises chest X-rays of all residents, including the homeless, at public health centres and in mobile units. Homeless people are encouraged to be screened at various facilities, such as shelters. Patients undergo chest X-ray screening during annual health checks and may also be screened in outpatient departments (OPDs) during visits for other diseases if the attending physician suspects TB or another respiratory disease. If any abnormal shadow is detected, further examinations are conducted, such as sputum smears for acid-fast bacilli, nucleic acid amplification, culture and computed tomography, mainly at Osaka Social Medical Center.

During 2015–2018, 92.1% (290/315) of PTB patients in Airin received daily DOT, whereby patients take anti-TB medicines daily in front of health care staff for the duration of treatment, in hospitals or in the community. (*3*)

The patient characteristics reported to increase the rate of TB case fatality in industrialized countries include demographic factors (increased age, male sex), social factors (homelessness), clinical aspects (positive sputum smear, multidrug-resistant TB, undernutrition/underweight, inadequate treatment, HIV infection) (*6*-*12*) and serious comorbidities such as those listed in the Charlson comorbidity index (17 conditions) or the Elixhauser score (30 comorbidities). (*13*, *14*)

The aim of this study was to guide interventions to reduce mortality from TB by determining the characteristics associated with mortality in patients with culture-positive PTB in Airin, Osaka City, between 2015 and 2018.

## Methods

### Study population

TB is a notifiable disease under the Infectious Disease Control Law in Japan; thus, physicians must report all TB cases to their local government. PTB patients registered in Airin between January 2015 and December 2018 were included in the study if they were alive at diagnosis, examined by chest X-ray and were culture-positive. Culture-positive PTB was defined as the presence of *Mycobacterium tuberculosis* in cultured sputum identified by immune-chromatography or nucleic acid amplification. Notifications of extrapulmonary TB were excluded, as most were not bacteriologically proven. Eligible PTB patients with coexisting extrapulmonary TB such as lymphadenitis and pleuritis were included.

Patients who moved out of Osaka City during the treatment period, in whom treatment failed (i.e. culture reversed from negative to positive or was persistently positive during treatment) or who were lost to follow-up were excluded from the analysis.

### Data collection

In routine practice, public health nurses (PHNs) at local public health centres record data on individual TB patients on structured patient cards, which are then entered onto an electronic spreadsheet. The data include sex, age, social condition (homeless or not), chest X-ray findings, bacteriological test results, mode of case detection and comorbidities. During interviews with the cases, PHNs also collect information such as height and body weight.

### Determination of death

In Japan, a TB patient’s attending physician is solely responsible for diagnosing the cause of death as TB-specific or non-TB-specific. In Osaka City, attending physicians and medical staff at the Public Health Office have monthly meetings at each hospital where TB patients are treated to discuss and agree on the cause of each TB patient’s death (TB or non-TB) for official records. Furthermore, by police request, any death of unknown cause identified in Osaka City is investigated by the Osaka Coroner’s Office, by inspection or autopsy.

### Variables

The outcome variables for this study were (1) treatment success: treatment completed and culture negative, “cured” for smear-positive patients and “treatment completion” for smear-negative patients; and (2) death: patients who died of any cause before or during treatment.

Possible explanatory variables were: sex (male, female); age (< 70, (*2*)70 years); homelessness (yes, no); cavity finding on chest X-ray (present, absent); bacteriological test results: sputum smear on Ziehl-Neelsen staining (positive, negative) and susceptibility to all five first-line anti-TB medicines, isoniazid, rifampicin, ethambutol, streptomycin and pyrazinamide (yes, no); body mass index (BMI) (< 18.5, (*2*)18.5 kg/m^2^); detection by “active screening” (yes, no), diabetes mellitus (present, absent); and other serious comorbidities (yes, no).

“Detection by active screening” was defined as diagnosis from a chest X-ray during active screening or at an outpatient visit for other diseases. This was compared with all other detection categories, e.g. at an outpatient visit for TB symptoms or during hospitalization for other diseases.

As diabetes mellitus was not shown to be associated with TB disease in a previous case–control study in Airin in 2015–2018, (*15*) this was analysed separately from other comorbidities.

### Analysis

To ascertain associations between the explanatory variables and mortality, univariate analysis was conducted with the χ^2^ test. Three separate analyses were conducted against treatment success: all deaths, early deaths (defined as death before or within the first 2 months of TB treatment) and late deaths (defined as death during the third month of treatment or later). The characteristics of TB-specific and non-TB-specific deaths were also compared. Variables with *P* < 0.1 were included in binomial multivariate logistic regression analysis, and adjusted odds ratios (aOR) were calculated.

Backward stepwise selection was applied to the binomial multivariate analysis. *P* < 0.05 was considered statistically significant. The univariate analysis was performed in Microsoft Excel® 2016, and binomial multivariate analysis was performed with SPSS version 11.0J for Windows (SPSS Inc., Chicago, IL, USA).

### Ethical considerations

The Ethical Review Committee of the Research Institute of Tuberculosis, Japan Anti-Tuberculosis Association, Tokyo, Japan, approved the study protocol (authorization number: RIT/IRB 2019–20). Informed consent was not obtained from eligible TB patients, as it is not required by the research ethics guidelines of the Japanese Government when research is conducted retrospectively from de-individualized, anonymous data collected routinely by legal requirement.

## Results

**Figure 1:**
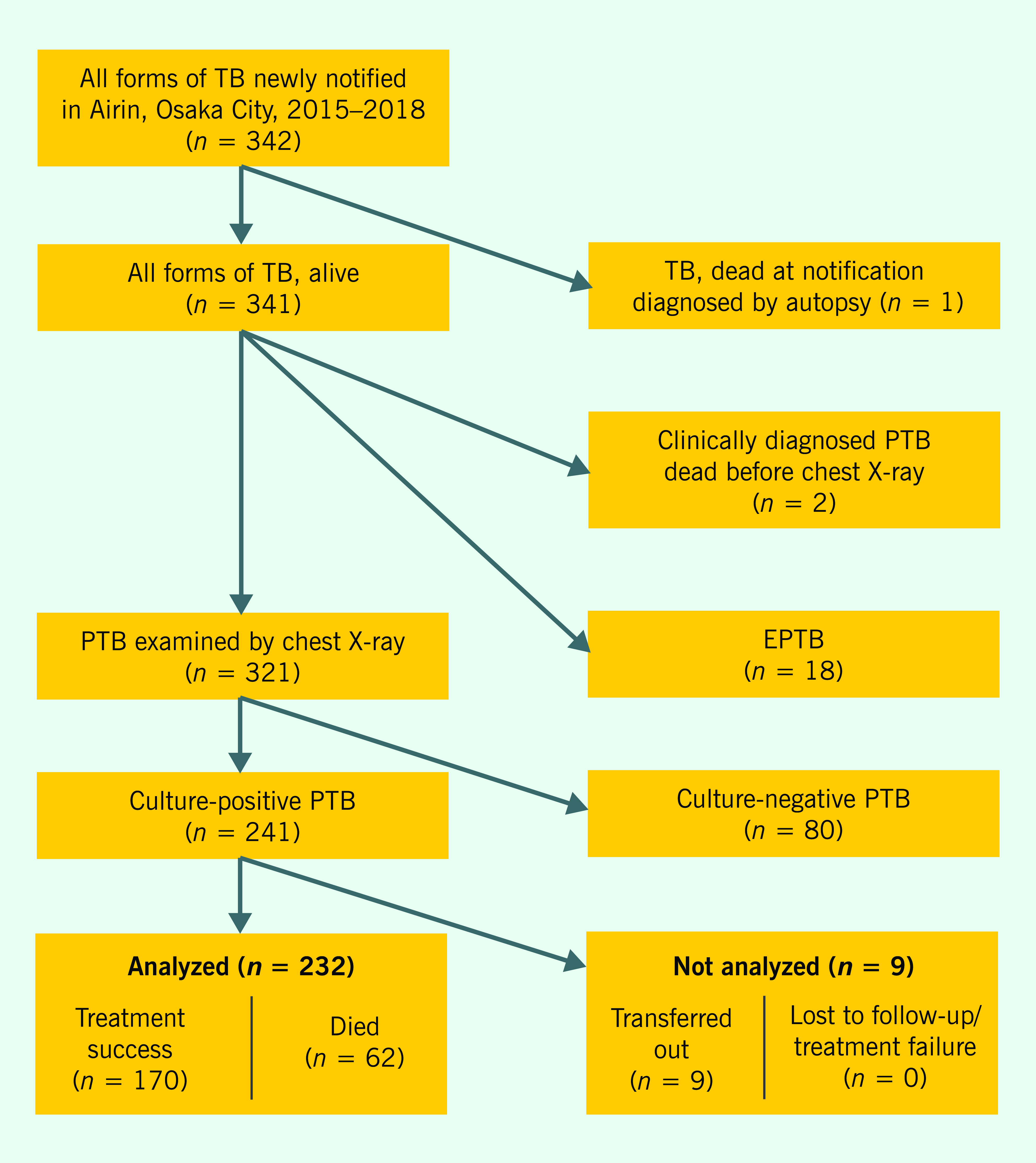
Flowchart of PTB case selection for analysis

Between January 2015 and December 2018, 342 TB patients were registered in Airin. One was diagnosed with TB at autopsy, two were clinically diagnosed and died before chest X-ray examination and 18 had extrapulmonary TB. Of the remaining 321 PTB patients who underwent a chest X-ray, 241 were culture-positive and 80 were culture-negative. Of the 241 culture-positive PTB cases, nine were transferred out of Osaka City, and none had treatment failure or was lost to follow-up. Therefore, 232 culture-positive PTB patients were included in the analysis (**Fig. 1**).

[**insert** Figure 1]

The mean age of the study participants was 66.4 years (range, 19–97), 95.7% (*n* = 222) were male, 51.9% (*n* = 121) were enrolled in PAP, and 29.2%  (*n* = 68) were homeless. There were two foreign-born patients.

The conditions included those listed in the Charlson comorbidity index and the Elixhauser comorbidity score as well as alcoholic pancreatitis, peritonitis, intestinal ileus and Parkinson disease (**Table 1**).

**Table 1:** Serious comorbidities included in the study of enrolled pulmonary tuberculosis patients, Airin, Osaka City, Japan, 2015–2018

Cancer	Affected organ: stomach, liver, lung, colon, bladder, larynx
Heart	Atrial fibrillation, acute cardiac ischaemia, cardiac bypass surgery, angina pectoris, chronic heart failure, dilated cardiomyopathy, sequelae of rheumatic fever
Lung	Pulmonary fibrosis, emphysema, chronic obstructive pulmonary disease, tracheostomy, asbestosis
Digestive organ	Hepatic disorder, alcoholic liver disorder, liver cirrhosis, hepatic failure, alcoholic pancreatitis, peritonitis, intestinal ileus, gastrostomy, gastrectomy
Kidney	Renal failure, nephrotic syndrome
Brain	Sequelae of cerebral infarction
Neurological disorder	Parkinson disease
Systemic	Anaemia, chronic thyroiditis, disuse syndrome

Treatment was completed by 170 patients, and 62 patients died before or during TB treatment, for a case fatality rate of 26.7%. There were 29 (46.7%) early deaths, including 3 (4.8%) before treatment, and 33 (53.2%) late deaths. Of the 59 patients who died during treatment, 55 (93.2%) were hospitalized from the beginning of treatment until death. Two were discharged from hospital but were readmitted when their general condition deteriorated just before death. Five died in the community.

The binomial multivariate analysis showed that, in comparison with treatment success, death of PTB patients was significantly associated with age (*2*)70 years, sputum smear positivity, underweight (BMI < 18.5) and presence of serious comorbidity. Detection of TB at screening was inversely associated with mortality. All these variables, apart from sputum smear positivity, were also significantly associated with early death in PTB patients. In the comparison of treatment success and late deaths of PTB patients, positive sputum smear and serious comorbidity were significantly associated with late death, and detection by TB screening was inversely associated with late death (**Table 2**).

**Table 2:** Univariate and multivariate analyses of characteristics associated with all deaths, early deaths and late deaths in pulmonary tuberculosis patients in Airin, Osaka City, 2015–2018

Characteristic	Treatment success (*n* = 170) *n*(%)	All deaths (*n* = 62)				Early deaths (*n* = 29)				Late deaths (*n* = 33)			
		*n*(%)	Univariate analysis *P*	Multivariate analysis aOR (95% CI)	*P*	*n*(%)	Univariate analysis *P*	Multivariate analysis aOR (95% CI)	*P*	*n*(%)	Univariate analysis *P*	Multivariate analyis aOR (95% CI)	*P*
**Sex**													
Male	165 (97.1)	57 (91.9)	0.089	0.19 (0.02–1.55)	0.122	28 (96.6)	0.883	-	-	29 (87.9)	0.019	0.11 (0.01–1.01)	0.051
Female	5 (2.9)	5 (8.1)	-	-	-	1 (3.4)	-	-	-	4 (12.1)	-	-	-
**Age**													
^3^70	53 (31.2)	37 (59.7)	< 0.001	2.66 (1.11–6.35)	0.027	19 (65.5)	< 0.001	4.39 (1.32–14.63)	0.016	18 (54.5)	0.010	2.01 (0.73–5.54)	0.176
< 70	117 (68.8)	25 (40.3)	-	-	-	10 (34.5)	-	-	-	15 (45.5)	-	-	-
**Country of birth**													
Japan	168 (98.8)	62 (100)	0.391	-	-	29 (100)	0.557	-	-	33 (100)	0.532	-	-
Other	2 (1.2)	0	-	-	-	0 (0)	-	-	-	0 (0)	-	-	-
**Homeless**													
Yes	54 (31.8)	14 (22.6)	0.174	-	-	6 (20.7)	0.230	-	-	8 (24.2)	0.402	-	-
No	116 (68.2)	48 (77.4)	-	-	-	23 (79.3)	-	-	-	25 (75.8)	-	-	-
**Cavity on chest X-ray**													
Yes	55 (32.4)	29 (46.8)	0.043	1.97 (0.81–4.80)	0.134	15 (51.7)	0.043	3.06 (0.90–10.46)	0.073	14 (42.4)	0.254	-	-
No	115 (67.6)	33 (53.2)	-	-	-	14 (48.3)	-	-	-	19 (57.6)	-	-	-
**Sputum smear**													
Positive	107 (62.9)	55 (88.7)	< 0.001	5.03 (1.51–16.80)	0.008	25 (86.2)	0.014	3.82 (0.79–18.32)	0.094	30 (90.9)	0.002	7.79 (1.53–39.76)	0.013
Negative	63 (37.1)	7 (11.3)	-	-	-	4 (13.7)	-	-	-	3 (9.1)	-	-	-
**Susceptibility to isoniazid, rifampicin, ethambutol, streptomycin and pyrazinamide**													
Yes	150 (88.2)	55 (88.7)	0.921	-	-	25 (86.2)	0.757	-	-	30 (90.9)	0.657	-	-
No	20 (11.8)	7 (11.3)	-	-	-	4 (13.8)	-	-	-	3 (9.1)	-	-	-
**Body mass index (kg/m^2^)**													
< 18.5	49 (29.5)	31 (59.6)	< 0.001	2.77 (1.17–6.53)	0.020	14 (63.6)	0.001	3.33 (1.03–10.72)	0.044	17 (56.7)	0.004	2.66 (0.98–7.25)	0.056
^3^18.5	117 (70.5)	21 (40.4)	-	-	-	8 (36.4)	-	-	-	13 (43.3)	-	-	-
Unknown	4	10	-	-	-	7	-	-	-	3	-	-	-
**Diabetes mellitus**													
Yes	37 (21.8)	12 (20.3)	0.818	-	-	5 (18.5)	0.702	-	-	7 (21.9)	0.989	-	-
No	133 (78.2)	47 (79.7)	-	-	-	22 (81.5)	-	-	-	25 (78.1)	-	-	-
Unknown	-	3	-	-	-	2	-	-	-	1	-	-	-
**Other serious comorbidity**													
Yes	41 (24.1)	38 (61.3)	< 0.001	5.56 (2.24–13.81)	< 0.001	18 (62.1)	< 0.001	6.15 (1.79–21.13)	0.004	20 (60.6)	< 0.001	6.45 (2.35–17.69)	< 0.001
No	129 (75.9)	24 (38.7)	-	-	-	11 (37.9)	-	-	-	13 (39.4)	-	-	-
**Detected by screening, including at outpatient department for other diseases**													
Yes	94 (55.3)	3 (4.8)	< 0.001	0.06 (0.02–0.24)	< 0.001	1 (3.4)	< 0.001	0.06 (0.01–0.49)	0.009	2 (6.1)	< 0.001	0.06 (0.01–0.32)	< 0.001
No	76 (44.7)	59 (95.2)	-	-	-	28 (96.6)	-	-	-	31 (93.9)	-	-	-

Of the 62 deaths, 35 were TB-specific and 27 non-TB-specific. Of the TB-specific deaths, 60.0% (21/35) were early deaths. In the multivariate analysis of patient characteristics for TB-specific and non-TB-specific deaths, TB-specific deaths were associated only with early death (aOR: 3.95, 95% confidence interval: 1.29; 12.07) (**Table 3**).

**Table 3:** Univariate and multivariate analyses of characteristics of TB-specific (*n* = 35) and non-TB-specific deaths (*n* = 27) among pulmonary TB patients, Airin, Osaka City, 2015–2018

Characteristic	TB-specific deaths *n*(%)	Non-TB-specific deaths *n*(%)	Univariate analysis *P*	Multivariate analysis aOR (95% CI)	*P*
**Sex**					
Male	35 (100%)	23 (85%)	0.019	-	-
Female	0 (0%)	4 (15%)	-	-	-
**Age (years)**					
^3^70	21 (60%)	16 (59%)	0.953	-	-
< 70	14 (40%)	11 (41%)	-	-	-
**Homeless**					
Yes	9 (26%)	5 (19%)	0.502	-	-
No	26 (74%)	22 (81%)	-	-	-
**Cavity on chest X-ray**					
Present	20 (57%)	9 (33%)	0.062	2.26 (0.73–6.94)	0.155
Absent	15 (43%)	18 (67%)	-	-	-
**Sputum smear**					
Positive	33 (94%)	22 (81%)	0.114	-	-
Negative	2 (6%)	5 (19%)	-	-	-
**Susceptible to isoniazid, rifampicin, ethambutol, streptomycin and pyrazinamide**					
Yes	31 (89%)	24 (89%)	0.969	-	-
No	4 (11%)	3 (11%)	-	-	-
**Body mass index**					
< 18.5	16 (59%)	15 (60%)	0.957	-	-
^3^18.5	11 (41%)	10 (40%)	-	-	-
Unknown	8	2	-	-	-
**Diabetes mellitus**					
Yes	5 (15%)	7 (27%)	0.265	-	-
No	28 (85%)	19 (73%)	-	-	-
Unknown	2	1	-	-	-
**Other serious comorbidity**					
Yes	18 (51%)	20 (74%)	0.070	0.32 (0.10–1.03)	0.057
No	17 (49%)	7 (26%)	-	-	-
**Detected by screening, including at outpatient department for other diseases**					
Yes	2 (6%)	1 (4%)	0.715	-	-
No	33 (94%)	26 (96%)	-	-	-
**Early death**					
Yes	21 (60%)	8 (30%)	0.017	3.95 (1.29–12.07)	0.016
No	14 (40%)	19 (70%)	-	-	-

## Discussion

The all-cause case fatality rate among culture-positive PTB patients in Airin, Osaka City, between 2015 and 2018 was 26.7%. Multivariate analysis showed that age (*2*)70 years, a positive sputum smear, BMI < 18.5 and serious comorbidity were associated with mortality in PTB patients. Detection by active screening or during an OPD visit for another disease was inversely associated with mortality in PTB patients. The frequency of characteristics did not differ between TB-specific and non-TB-specific deaths. Similar results were reported in a much larger national study in the USA, although different methods were used to determine TB-specific deaths. (*10*)

Of the fatal PTB cases in Airin, 93% were in a hospital from the beginning of treatment until death. Almost all (92%) of the cases during the study period received daily DOT in the Airin TB programme. None of the TB patients in the study failed treatment or were lost to follow-up; therefore, none of the deaths was due to treatment interruption or non-adherence, which may have contributed to deaths in other studies. (*8*)

About half of the fatalities among PTB patients were early deaths, occurring before or within the first 2 months of treatment. Similar findings were reported in Australia, (*6*) Denmark, (*12*) China, Taiwan (China), (*16*) Spain, (*17*) the Republic of Korea, (*18*) the United Kingdom (*19*) and Finland. (*20*) We also found that a higher proportion of early deaths were TB-specific rather than non-TB-specific, which suggests that patients who survive > 2 months of TB treatment have better outcomes, and late deaths are due more commonly to causes other than TB.

Advanced age is well recognized as a risk factor for morbidity and mortality from TB because of weakening of both the innate and adaptive immune systems. (*21*) The presence of comorbidity has also been identified as the most common characteristic of death from TB in other developed countries, such as Australia, (*6*) Denmark, (*12*) Finland, (*20*) the Netherlands, (*22*) Spain, (*17*) Singapore, (*23*) the Republic of Korea, (*18*) China, Taiwan (China) (*9*, *11*, *24*) and the USA. (*10*) Older people tend to be more vulnerable to comorbidity, as suggested by a one-day survey of the prevalence of morbidity in 2017 conducted by random stratified sampling in 6402 hospitals (76% of hospitals in Japan), which showed that the rate of comorbidity among people aged (*2*)65 years was 17 times higher than among those aged 20–24 years. (*25*) In our study, both older age and having a comorbidity were associated with PTB mortality.

A BMI of < 18.5 was significantly associated with all deaths and with early deaths of PTB patients in this study but not with late deaths of PTB patients. A similar finding was made in a study in China, Taiwan (China). (*16*) Undernutrition impairs cell-mediated immunity, which increases vulnerability to specific infectious diseases, including TB. (*26*, *27*) A BMI of < 18.5 defines underweight and is a good proxy indicator of undernutrition. A literature review of cohort studies in the USA, Europe, India, Bangladesh and East Asia showed that being underweight was associated with a significantly higher risk for all-cause mortality. (*28*)

In contrast, our study showed that having a positive sputum smear status was associated with all deaths and late deaths in PTB patients but not with early deaths in these patients. A review of individual patient records in our study showed that about half of smear-negative cases had treatment delays of about 2 months until the culture became positive; however, the other half of smear-negative cases had no delay in TB treatment, because other methods of sputum smear examination on the same day led to a diagnosis of TB, e.g. cases in which TB bacilli were confirmed by nucleic acid amplification or chest X-ray showed typical infiltration of TB. This may explain our findings.

Detection of TB by screening and during an OPD visit for other diseases were both considered early diagnoses because these patients were unlikely to have symptoms of TB. In each of our analyses, early diagnosis was inversely associated with mortality from PTB, in that fatal PTB cases had significantly smaller odds of being detected with these early diagnosis measures. A study in Norway showed that the case fatality rate of TB patients detected through passive case finding due to symptoms was 11.1% and that of immigrants, close contacts of infectious cases and others screened for TB was 6.2%, suggesting that patients who are symptomatic at diagnosis have a higher case fatality rate. (*29*) In Airin, high-risk patients have the opportunity to undergo TB screening, as medical services are available to all through universal health coverage via health insurance or PAP.

The limitations to this study were as follows. Smoking, alcohol use and initial TB symptoms were excluded from the analysis because of lack of data, as the critical condition of patients who died early obviated questioning by PHNs during interviews. Therefore, delayed TB diagnosis was not included in the analysis. HIV infection was not included because TB/HIV co-infection was diagnosed for only 0.4% (42/10 038) of TB patients in Osaka City in 2008–2016 (unpublished data). The severity of comorbidities was determined from reported diagnoses, with no detailed clinical or laboratory test results. Finally, as a special chest X-ray screening programme is available only in Airin, as mentioned above, this study may not be generalizable to other parts of Osaka City.

## Conclusion

Old age, severe disease, undernutrition and serious comorbidities were associated with mortality of PTB patients. Detection of PTB by chest X-ray screening and regular visits to OPDs for other diseases was associated with non-fatal TB, perhaps because they contributed to early case finding. Therefore, current active TB case finding and health education on regular visits to physicians for any disease should be further strengthened in the urban poor setting in Osaka City, Japan. Attending physicians should be advised to take periodic chest X-rays for aged patients with serious comorbidities or low BMI, regardless of symptoms and even in other parts of Osaka City if the facilities are available.
